# Infective Endocarditis by Biofilm-Producing Methicillin-Resistant *Staphylococcus aureus*—Pathogenesis, Diagnosis, and Management

**DOI:** 10.3390/antibiotics13121132

**Published:** 2024-11-25

**Authors:** Ashlesha Kaushik, Helen Kest, Mangla Sood, Corey Thieman, Bryan W. Steussy, Michael Padomek, Sandeep Gupta

**Affiliations:** 1 Division of Pediatric Infectious Diseases, Unity Point Health at St. Luke’s Regional Medical Center and University of Iowa Carver College of Medicine, 2720 Stone Park Blvd, Sioux City, IA 51104, USA; 2 Division of Pediatric Infectious Diseases, St. Joseph’s Children’s Hospital, 703 Main Street, Paterson, NJ 07503, USA; kesth@sjhmc.org; 3 Department of Pediatrics, Indira Gandhi Medical College, Shimla 171006, HP, India; drmanglasood@gmail.com; 4 Division of Pharmacology, Unity Point Health at St. Luke’s Regional Medical Center, 2720 Stone Park Blvd, Sioux City, IA 51104, USA; corey.thieman@unitypoint.org (C.T.); michael.padomek@unitypoint.org (M.P.); 5 Division of Microbiology, Unity Point Health at St. Luke’s Regional Medical Center, 2720 Stone Park Blvd, Sioux City, IA 51104, USA; bryan.steussy@unitypoint.org; 6 Division of Pulmonary and Critical Care, Unity Point Health at St. Luke’s Regional Medical Center, 2720 Stone Park Blvd, Sioux City, IA 51104, USA; sandeep.gupta@unitypoint.org

**Keywords:** methicillin-resistant *Staphylococcus aureus*, MRSA, biofilm, infective endocarditis, antibiotics, treatment

## Abstract

Infective endocarditis (IE) is a life-threatening condition with increasing global incidence, primarily caused by *Staphylococcus aureus*, especially methicillin-resistant strains (MRSA). Biofilm formation by *S. aureus* is a critical factor in pathogenesis, contributing to antimicrobial resistance and complicating the treatment of infections involving prosthetic valves and cardiovascular devices. Biofilms provide a protective matrix for MRSA, shielding it from antibiotics and host immune defenses, leading to persistent infections and increased complications, particularly in cases involving prosthetic materials. Clinical manifestations range from acute to chronic presentations, with complications such as heart failure, embolic events, and neurological deficits. Diagnosis relies on the Modified Duke Criteria, which have been updated to incorporate modern cardiovascular interventions and advanced imaging techniques, such as PET/CT (positron emission tomography, computed tomography), to improve the detection of biofilm-associated infections. Management of MRSA-associated IE requires prolonged antimicrobial therapy, often with vancomycin or daptomycin, needing a combination of antimicrobials in the setting of prosthetic materials and frequently necessitates surgical intervention to remove infected prosthetic material or repair damaged heart valves. Anticoagulation remains controversial, with novel therapies like dabigatran showing potential benefits in reducing thrombus formation. Despite progress in treatment, biofilm-associated resistance poses ongoing challenges. Emerging therapeutic strategies, including combination antimicrobial regimens, bacteriophage therapy, antimicrobial peptides (AMPs), quorum sensing inhibitors (QSIs), hyperbaric oxygen therapy, and nanoparticle-based drug delivery systems, offer promising approaches to overcoming biofilm-related resistance and improving patient outcomes. This review provides an overview of the pathogenesis, current management guidelines, and future directions for treating biofilm-related MRSA IE.

## 1. Introduction

Infective endocarditis (IE) is a severe, life-threatening condition characterized by infection of the endocardial surface of the heart, including the heart valves. Despite advances in diagnostic techniques and treatment, the global incidence of IE has been steadily increasing, and IE is ranked among the five most common life-threatening infectious conditions resulting in significant morbidity and mortality [1,2,3,4]. The epidemiology of IE has evolved over recent decades, with *Staphylococcus aureus*, particularly methicillin-resistant *Staphylococcus aureus* (MRSA), now emerging as the leading causative pathogen, surpassing traditional culprits like *Streptococcus* species [2]. The rising prevalence of *S. aureus*-related IE is partly attributed to the increasing use of cardiovascular interventions, such as prosthetic valves, cardiac implants, and central venous catheters, which create surfaces conducive to bacterial colonization and biofilm formation [1,5,6]. This shift has contributed to the complexity of IE cases, leading to increased mortality and morbidity despite medical advancements [6,7].

Biofilm formation plays a central role in the pathogenesis of *S. aureus* infections [8]. Biofilms are complex, multicellular communities of bacteria encased in a self-produced extracellular polymeric substance (EPS), which includes polysaccharides, proteins, lipids, and extracellular DNA [9,10,11]. This protective matrix allows *S. aureus* to evade host immune defenses and withstand antimicrobial agents, resulting in persistent and recurrent infections, particularly on native and prosthetic cardiac surfaces [7,11]. The ability of *S. aureus* to form biofilms is regulated by a complex genetic network involving components such as Microbial Surface Components Recognizing Adhesive Matrix Molecules (MSCRAMMs), the *ica* locus, the *agr* system, and *cid/lrg* networks [12,13,14]. Additionally, various host factors, including endothelial cell receptors, platelets, and subendothelial matrix proteins, facilitate bacterial adherence to cardiac tissues, thereby enhancing biofilm formation and contributing to the development of IE [15]. These mechanisms underscore the difficulty of eradicating biofilm-associated infections with standard antimicrobial therapies alone [7,16].

Clinically, IE presents with a spectrum of symptoms ranging from acute, rapidly progressive illness to subacute or chronic conditions with more subtle manifestations. The associated complications, including valvular damage, heart failure, systemic embolization, and neurological deficits, contribute to the high morbidity and mortality rates associated with the disease [1,2]. Early diagnosis and intervention are crucial for improving outcomes. The Modified Duke Criteria, which incorporate clinical, microbiological, and imaging findings, remain the gold standard for diagnosing IE [17,18]. However, the evolving landscape of cardiac care—particularly with the increased use of prosthetic valves and implantable devices—has necessitated updates to the diagnostic criteria to include imaging and diagnostic considerations for device-associated infections [18,19].

The management of IE, especially biofilm-associated MRSA infections, presents unique challenges. The cornerstone of therapy involves prolonged antimicrobial treatment, typically with agents such as vancomycin or daptomycin, often in combination with surgical intervention to remove infected tissue or prosthetic material when necessary [1,2,20,21,22]. However, the protective nature of biofilms and the emergence of antibiotic resistance complicate therapy, often leading to treatment failures and relapses [7]. Additionally, the role of anticoagulation in IE remains controversial due to the risk of hemorrhage, although emerging adjunctive therapies like dabigatran have shown potential benefits in mitigating complications [1,23].

Given the challenges in treating biofilm-associated infections, novel therapeutic strategies are being explored [24,25,26,27,28,29,30,31]. These include combination antibiotic regimens, bacteriophage therapy, hyperbaric oxygen therapy, and the use of agents like N-acetylcysteine to disrupt biofilm integrity [24,25,26,27,30,31]. Advancements in nanoparticle-based drug delivery and surface-modifying agents also offer potential avenues for enhancing antimicrobial efficacy and biofilm disruption [26,27,31]. This review aims to provide a comprehensive analysis of the pathogenesis of biofilm-associated *S. aureus* infections in IE, explore the clinical manifestations and complications, and discuss current therapeutic guidelines alongside potential novel interventions aimed at improving patient outcomes.

## 2. Pathogenesis

The pathogenesis of MRSA in infective endocarditis is primarily driven by its ability to form biofilms, a critical factor in its resistance to antimicrobial therapies and host immune defenses. The formation and maintenance of biofilms are governed by a complex genetic network involving microbial surface components recognizing adhesive matrix molecules (MSCRAMMs), the *ica* locus, the *cid/lrg* network, *tar* genes, *codY*, *sarA*, the *agr* system, and the *sae* two-component systems (TCS) [8,13,14]. In the early stages of infection, biofilm-related genes (e.g., *ica* locus, *cid/lrg* network, and *tar* genes) and colonization genes (e.g., microbial surface components recognizing adhesive matrix molecules) are upregulated, enabling MRSA to adhere to host tissues and establish infection [8,32].

Host factors also play a crucial role in MRSA’s ability to colonize endothelial surfaces. Subendothelial matrix proteins, endothelial cell receptors, and platelets interact with MRSA cell wall adhesins, such as fibronectin-binding proteins, promoting bacterial attachment to these surfaces and enhancing biofilm formation [33]. This interaction between MRSA and host components is pivotal in the development of IE.

MRSA biofilm formation in IE follows a multi-step process: attachment, maturation, and dispersal [34]. The attachment stage is particularly significant in MRSA-associated IE, which accounts for about 30% of IE cases worldwide [7]. Two key pathways facilitate attachment: the damage-induced pathway and the inflammation-induced pathway [15]. In the damage-induced pathway, pre-existing valve damage leads to the deposition of fibrinogen on the valve surface, which MRSA exploits using clumping factor A (ClfA) to adhere to fibrinogen [35]. ClfA and clumping factor B (ClfB), among the primary MSCRAMMs, help MRSA adhere to biotic surfaces like heart valves and abiotic surfaces such as prosthetic devices [9].

In contrast, the inflammation-induced pathway does not rely on ClfA but involves endothelial cell activation due to inflammation. This process promotes platelet adhesion, which entraps MRSA on the valve surface, allowing IE to develop even in the absence of prior valve damage [15].

Following attachment, MRSA biofilms undergo maturation. During this stage, MRSA secretes an extracellular polymeric substance (EPS), primarily composed of polysaccharide intercellular adhesin (PIA), encoded by the *ica* operon [9,10]. PIA is integral to bacterial accumulation within the biofilm. In IE, platelet clumps and neutrophil extracellular traps (NETs) contribute to the formation of vegetation, trapping bacteria within a matrix of neutrophils and platelets [16,36]. The mature biofilm structure consists of bacterial colonies surrounded by fibrin and platelets [37]. This protective EPS matrix not only shields the bacteria from host immune defenses but also impedes antibiotic penetration, complicating treatment [7,12,38,39].

As biofilms mature, increasing bacterial density depletes nutrients and oxygen, relieving repression by CodY, a global transcriptional regulator of virulence genes. This shift upregulates systems such as *agr* and *sae* TCS, leading to toxin production that aids in nutrient acquisition, immune evasion, and further dissemination of infection [40]. MRSA toxins such as alpha-toxin, Panton–Valentine leukocidin (PVL), and others are released in response to these changes, exacerbating tissue damage and immune dysregulation. These toxins further fuel the inflammatory response, contributing to immune system exhaustion and organ dysfunction [38].

In severe cases, this dysregulated immune response can lead to a cytokine storm—an overwhelming release of pro-inflammatory cytokines like IL-6, IL-1β, and TNF-α. This hyperinflammatory state, often triggered by persistent infection and biofilm formation, results in widespread tissue damage, endothelial disruption, and immune cell exhaustion, exacerbating the disease process [39,40]. The cytokine storm, combined with ongoing bacterial dissemination and toxin release, may lead to septic shock, systemic inflammation, and multi-organ failure. If left unchecked, this cascade can result in death, particularly in patients with underlying comorbidities or weakened immune systems [38,39].

Biofilm dispersal occurs through both mechanical forces and enzymatic degradation of the EPS. Dispersal allows parts of the biofilm to spread and colonize new areas within the host [10]. This process is regulated by the accessory gene regulatory (*agr*) system, encoding AgrA, AgrB, AgrC, and AgrD. This system responds to environmental signals like nutrient and oxygen levels, toxin concentrations, and other stressors [41]. Quorum sensing, a cell-to-cell communication system mediated by *agr*, can trigger biofilm dispersal by accumulating autoinducing peptides (encoded by *agrD*), which are potential therapeutic targets for biofilm disruption [42].

Dispersal of MRSA biofilms can occur actively or passively. Active dispersal involves the bacterial secretion of enzymes, such as phosphodiesterases, which degrade the biofilm matrix. Passive dispersal, on the other hand, occurs through mechanical forces or external enzymatic breakdown [12]. In MRSA-associated IE, biofilm dispersal frequently manifests as septic emboli, where fragments of vegetation break off and block downstream vessels, leading to embolic events in up to 50% of cases [43]. These emboli can travel to various organs, including the lungs in right-sided IE or the brain in left-sided IE. This process is exacerbated by bacterial degradation of the biofilm, which weakens the matrix and facilitates the detachment of fragments [33,44].

**Figure 1:**
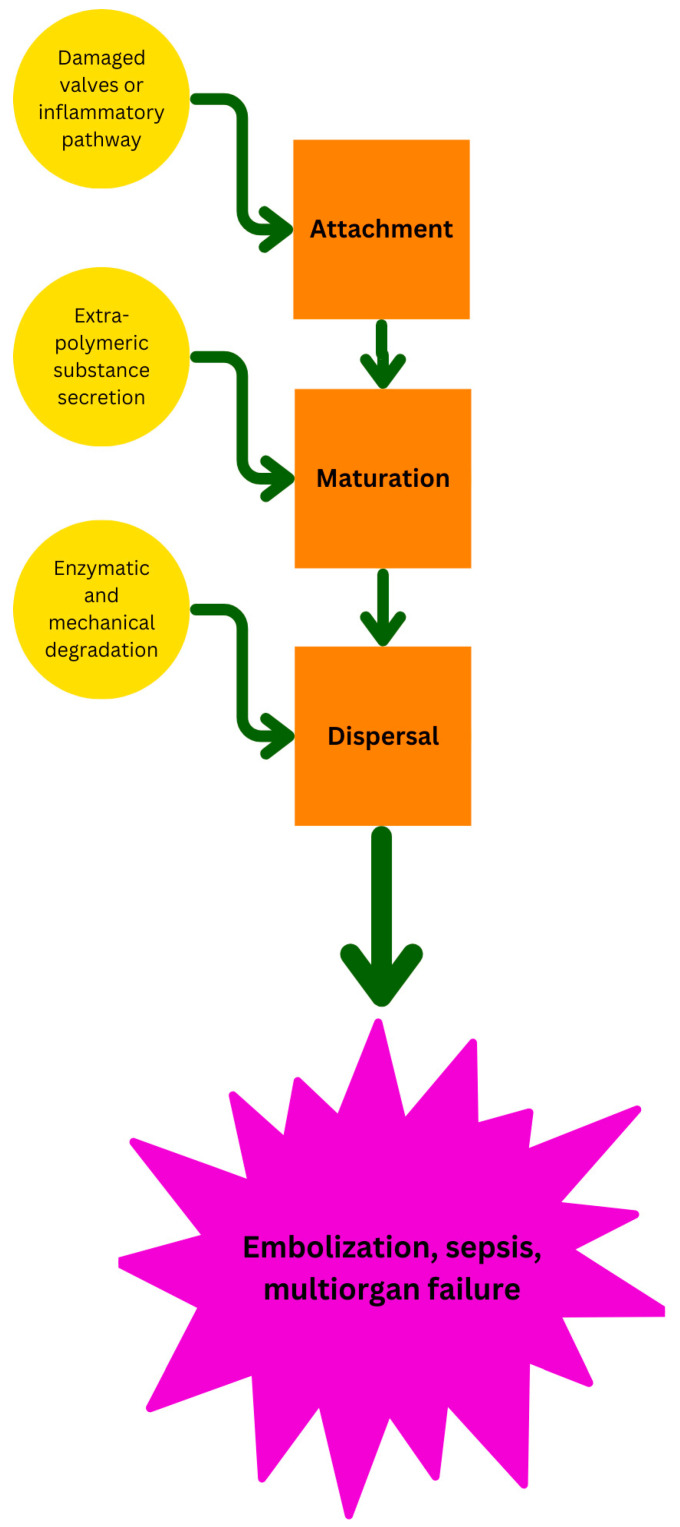
Stages in biofilm formation in MRSA IE. Stages are represented in orange boxes. Facilitating factors for each stage are shown in yellow boxes.

Septic emboli, along with the internalization of MRSA into endothelial cells, create environments where antibiotics and host immune responses have limited access to the pathogen. As the infection spreads through the bloodstream and tissues, unchecked immune responses can further escalate, resulting in systemic inflammation and septic shock. This can lead to life-threatening complications, including multi-organ failure and death, especially in cases where timely intervention is not achieved [38]. Stages of biofilm formation in the pathogenesis of MRSA IE are schematically depicted in Figure 1. Pathogenesis of MRSA IE is summarized in Table 1.

## 3. Clinical Manifestations, Complications, and Diagnosis of Infective Endocarditis

Infective endocarditis can present with a wide range of clinical symptoms, from acute, rapidly progressive illness to a more subacute or chronic condition with subtle signs. Given this variability, a high index of suspicion is crucial, particularly in patients with unexplained fever [1,2]. Early diagnosis is essential to prevent complications, which can significantly impact patient outcomes.

### 3.1. Clinical Manifestations

Fever is the most commonly reported symptom, present in approximately 90% of patients, often accompanied by chills and weight loss [1,2,45]. Continuous bacteremia is a hallmark of IE, with fever and positive blood cultures being key diagnostic features [1,2]. Other common symptoms include myalgias, arthralgias, anorexia, malaise, and night sweats [1,2,45]. On physical examination, a new or changing cardiac murmur is a classic sign, observed in about 85% of patients. Additional findings may include splenomegaly, petechiae, and splinter hemorrhages under the nail beds.

Rare manifestations of IE include Janeway lesions (erythematous lesions on the palms and soles), Osler nodes (painful subcutaneous nodules on the digits), and Roth spots (hemorrhagic retinal lesions with pale centers) [46,47]. Roth spots occur in less than 5% of cases [48]. Janeway lesions are more commonly associated with acute IE, while Osler nodes and Roth spots tend to be seen in subacute or chronic forms of the disease [46,47].

Data from the European Infective Endocarditis Registry (EURO-ENDO) indicate that fever (77.7%), cardiac murmur (64.5%), and congestive heart failure (27.2%) are the most frequently recorded clinical manifestations [2,49]. About 25% of patients experience embolic complications, while 11.5% develop cardiac conduction abnormalities [2,49]. These manifestations underscore the complexity and potential severity of IE, particularly in cases associated with biofilm formation on cardiac tissues or prosthetic devices.

### 3.2. Complications

IE can lead to several serious, life-threatening complications. Cardiac complications are the most common, occurring in up to 50% of patients. These include valvular regurgitation, heart failure, and conduction abnormalities such as heart block [1,50]. The presence of vegetation on heart valves can lead to valve destruction, resulting in hemodynamic instability and the potential need for surgical intervention.

Neurological complications occur in approximately 40% of patients and include stroke, brain abscess, and intracranial hemorrhage [1,2,45]. Embolic events, resulting from fragments of infected vegetation breaking off and traveling to other parts of the body, are a significant concern.

Septic emboli can lead to renal and splenic infarctions, septic pulmonary emboli, and metastatic infections such as vertebral osteomyelitis, septic arthritis, and splenic abscesses [1,2,45]. These embolic phenomena are particularly common in cases involving *Staphylococcus aureus*, known for its aggressive nature and tendency to form biofilms.

*S. aureus*-associated IE primarily affects the left side of the heart in non-intravenous drug users, with high mortality rates ranging from 25% to 40% [1]. In contrast, intravenous drug users are more prone to tricuspid valve involvement, and cure rates for right-sided *S. aureus* IE exceed 85% [1]. The presence of biofilms on prosthetic materials and devices further complicates treatment, often leading to persistent infections and an increased risk of systemic embolization.

### 3.3. Diagnosis of IE

Diagnosis of IE requires a combination of clinical, microbiological, and imaging evidence. The Modified Duke Criteria, initially proposed in 1994 and later updated, remain the gold standard for diagnosing IE [17,18]. This classification stratifies patients into “definite”, “possible”, or “rejected” categories based on major and minor criteria, which include clinical findings, blood culture results, and echocardiographic evidence. The detailed criteria are presented in Box A1 and Box A2.

Given recent advances in cardiovascular interventions, including prosthetic valves and implantable devices, the diagnosis of IE has become more complex. The 2023 Duke International Society for Cardiovascular Infectious Disease (ISCVID) guidelines introduced modifications to the Duke Criteria to account for prosthetic devices and endovascular intracardiac implantable electronic devices (CIEDs), which are associated with a higher risk of biofilm-related infections [19]. These updates include the following:

1. Pathological Criteria: The addition of microorganisms identified from explanted prosthetic valves, sewing rings, ascending aortic grafts (with concomitant valve involvement), CIEDs, or arterial emboli [19].
2. Imaging Major Criteria: Cardiac CT with echocardiography and metabolic activity on PET/CT imaging of prosthetic materials, such as valves, grafts, and intracardiac leads, are now part of the diagnostic process.
3. Surgical Major Criteria: Direct inspection evidence of IE during heart surgery is included to enhance diagnostic accuracy [19].
4. Minor Criteria: Inclusion of metabolic abnormalities on imaging within 3 months of prosthetic material implantation [19].

These modifications underscore the evolving landscape of IE diagnosis, particularly emphasizing the role of advanced imaging techniques and the increased awareness of biofilm-associated infections, especially in patients with cardiovascular implants. Recent studies have validated these updates [51,52]. Incorporating these advancements into clinical practice is crucial for the early detection and effective management of IE.

## 4. Approach to Treatment of IE

The treatment of infective endocarditis requires a multifaceted approach, combining targeted antimicrobial therapy with surgical intervention, particularly for cases complicated by biofilm-producing pathogens like *Staphylococcus aureus*. Management strategies are guided by clinical presentation, microbiological data, and the presence of native or prosthetic cardiac material. Despite advancements in antimicrobial therapy, biofilm formation and the consequent antimicrobial resistance continue to present major challenges in managing these infections [7]. Prolonged treatment with high-dose antibiotics, often utilizing combination therapies with different mechanisms of action, plays a crucial role [1]. Studies have shown that early-stage biofilms are more susceptible to antibiotic treatment than fully mature biofilms [53], underscoring the importance of early and aggressive antibiotic intervention to enhance treatment effectiveness. In addition to antibiotic therapy, early surgical intervention is frequently required to achieve complete clearance of the infection [1].

### 4.1. Treatment of Methicillin-Resistant Staphylococcus aureus (MRSA) Infective Endocarditis (IE)

#### 4.1.1. Antimicrobial Therapy

Antibiotic therapy remains the backbone of IE management, with treatment regimens tailored to the specific organism and its susceptibility profile. The treatment of MRSA-related IE is particularly challenging due to the pathogen’s biofilm formation and inherent resistance to multiple antibiotics. This section describes the antimicrobial treatment recommendations for MRSA IE in adult and pediatric populations in accordance with American Heart Association (AHA), Infectious Diseases Society of America (IDSA), and European Society of Cardiology (ESC) guidelines [1,2]. These guidelines also recommend the drainage of abscesses and removal of infected devices to enhance the efficacy of antimicrobial agents [1,2].

MRSA endocarditis treatment recommendations differ considering whether the patient is exhibiting native valve or prosthetic valve endocarditis (PVE). Detailed IE treatment regimens are presented in Table 2 [1,2], with pharmacokinetic properties and dosages for antimicrobials used in MRSA IE treatment outlined in Table 3 and Table 4 [1,2].

##### Native Valve Endocarditis (NVE)

First-Line Therapy: Vancomycin is the drug of choice for MRSA NVE [1,2]. According to the 2023 European Society of Cardiology (ESC) guidelines, both right-sided and left-sided NVE caused by MRSA in adults should be treated with intravenous vancomycin, aiming for an area under the curve (AUC) of 400–600 mg·h/L over a 6-week period [2]. If vancomycin is contraindicated or if the MRSA isolate has a minimum inhibitory concentration (MIC) > 1 mg/L, daptomycin is recommended as an alternative. In most cases, intravenous therapy is advised for the entire treatment duration.

Second-Line Agents: In patients intolerant to vancomycin, daptomycin combined with ceftaroline, cloxacillin, or fosfomycin (availabel orally in some regions) is suggested [2]. Combination therapy not only enhances bacterial clearance but also reduces the risk of resistance development. High-dose daptomycin (10 mg/kg) for a 6-week duration is favored to mitigate the risk of resistance. While combination therapy is preferred, rifampin and gentamicin are generally avoided due to their potential for hepatotoxicity and nephrotoxicity and limited evidence for improved survival in this population.

Pediatric NVE: For children, the American Heart Association (AHA)/IDSA guidelines recommend vancomycin as the first-line agent. In contrast to adult protocols, pediatric patients may receive synergistic gentamicin for the first 3–5 days of therapy to enhance bacterial killing. Intravenous therapy should continue for the full 6-week duration following the first negative blood culture [1].

##### Prosthetic Valve Endocarditis (PVE)

PVE poses additional treatment challenges due to the presence of prosthetic material, which fosters biofilm formation and increases the risk of antimicrobial resistance. The ESC and AHA/IDSA guidelines recommend a minimum of 6 weeks of parenteral antibiotics.

First-Line Therapy: Vancomycin in combination with gentamicin and rifampin is recommended for PVE caused by MRSA. This combination is crucial because rifampin’s biofilm penetration enhances the treatment’s efficacy [1,2]. While nafcillin or oxacillin are used alongside rifampin for methicillin-susceptible *S. aureus* (MSSA) PVE, vancomycin and rifampin are used for MRSA. Gentamicin should be administered for the first 2 weeks to limit nephrotoxicity related to prolonged use.

For MRSA isolates with a vancomycin MIC of 1 mg/L or less, a goal AUC of 400–600 mg*h/L is optimal for vancomycin [54,55]. Concomitant therapy with gentamicin should be initiated with vancomycin on the first day, and a synergistic dose of 1 mg/kg every 8 h should be utilized [1,2,55]. For synergy, recommended goal gentamicin peaks are lower than conventional therapy at 3–4 mcg/mL. Goal troughs for gentamicin are ideally <1 mcg/mL. Peak and trough levels should be obtained at steady state and drawn with the 3rd dose of gentamicin. When treating isolates resistant to gentamicin, an alternative aminoglycoside (e.g., tobramycin) can be used if the isolate shows susceptibility. Rifampin should be initiated parenterally at a dose of 900–1200 mg/24 h given in 2–3 divided doses [1,2].

Second-Line Therapy for PVE: For patients unable to take vancomycin, daptomycin combined with gentamicin and rifampin is the preferred second-line option. For those at high risk of nephrotoxicity, a regimen of daptomycin, ceftaroline, and rifampin may be used. Although optimal dosing of ceftaroline for endocarditis has not been firmly established, the ESC recommends 600 mg every 8 h [2,56].

**Figure 2:**
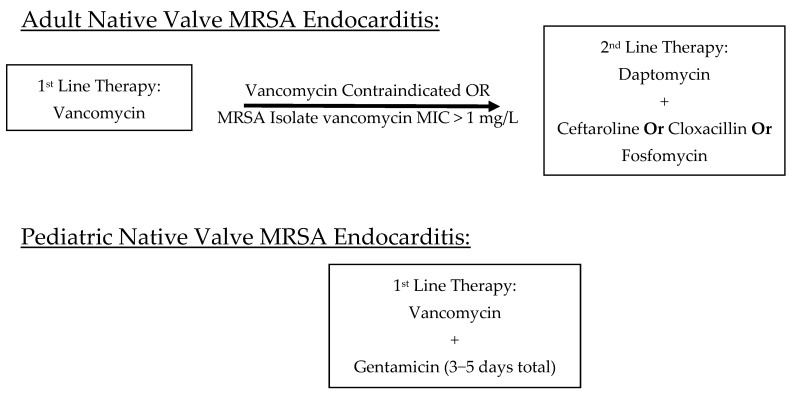

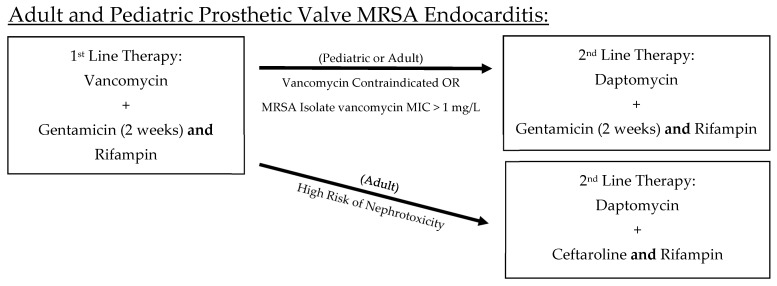
Schematic representation of treatments for native valve and prosthetic valve MRSA IE [1,2].

Pediatric PVE: Treatment regimens for PVE in pediatric patients are similar to adults. The AHA/IDSA Guidelines recommend concomitant therapy with vancomycin and rifampin [5 mg/kg every 8 h (maximum dose 900 mg/24 h)] for at least 6 weeks with synergistic gentamicin (1 mg/kg every 8 h) for 2 weeks [1]. First-line and second-line therapies for native valve and prosthetic valve MRSA IE are schematically presented in Figure 2.

#### 4.1.2. Surgical Management

Surgical intervention is a pivotal aspect of managing infective endocarditis (IE), especially in cases where prosthetic materials are involved or when biofilm formation renders antibiotic therapy insufficient. Biofilms form a formidable barrier against antibiotics, drastically reducing drug penetration and fostering persistent infection. As a result, surgery is often the only definitive solution to clear the infection, remove biofilm-laden tissues, and prevent further complications. In cases of severe valvular destruction, heart failure, or unrelenting infection, surgical intervention becomes not just an option but a necessity for patient survival.

It is estimated that nearly 50% of all IE cases require heart valve surgery, driven by critical issues such as extensive valvular damage, large vegetation, or failure of antibiotic therapy to control the infection [57,58]. The primary goals of surgery include repairing or replacing damaged valves, excising infected tissue, and removing prosthetic materials that are sources of biofilm. The timing of the surgery is crucial, with early intervention often significantly improving outcomes, particularly in cases complicated by heart failure, embolic risk, or uncontrolled infection. Without prompt surgical management, the risk of recurrent infection and mortality increases, underscoring the essential role of surgery in the comprehensive treatment of IE.

Prompt surgical removal of infected devices is crucial for biofilm-related infections since the presence of a foreign body reduces the effectiveness of antibiotics and impedes phagocytosis [1,53].

Indications for Surgery: Per AHA/IDSA guidelines, surgery is indicated in patients with heart failure caused by valve dehiscence, intracardiac fistula, or severe prosthetic valve dysfunction. It is also recommended for persistent bacteremia despite 5–7 days of appropriate antibiotics, recurrent embolic events, relapsing PVE, or mobile vegetation larger than 10 mm [1]. Surgery is essential in cases complicated by heart block, annular or aortic abscess, or destructive lesions [1].

Timing of Surgery in Stroke: For patients with stroke or cerebral emboli, valve surgery can proceed if intracranial hemorrhage has been ruled out. However, a delay of at least 4 weeks is recommended for patients with major ischemic stroke or intracranial hemorrhage [1].

#### 4.1.3. Anticoagulation

The use of anticoagulation in IE is controversial due to the heightened risk of hemorrhage. Despite the thrombotic complications that patients with infective endocarditis and prosthetic heart valves are predisposed to, no additional anticoagulation has been shown to provide benefit in this population given an increased propensity of hemorrhagic complications. If patients are currently taking an anticoagulant for a specific indication, the risk and benefits should be weighed, and generally the patient should continue the medication. Patients with prosthetic valves should continue their vitamin K antagonist therapy, with INR goals tailored to valve type and location (e.g., 3.0–3.5 for mitral valves, 2.0–3.0 for aortic valves). The IDSA guidelines recommend discontinuing anticoagulation for at least 2 weeks in patients with mechanical valve IE who have experienced a central nervous system embolic event [1]. Aspirin as an adjunctive therapy is not recommended due to its lack of demonstrated benefit [1].

Interestingly, the direct thrombin inhibitor dabigatran has shown potential in reducing thrombus formation in MRSA IE by inhibiting *staphylocoagulase*-mediated clotting. All *Staphylococcus aureus* isolates express *staphylocoagulase*, which leads to coagulation cascade activation, causing thrombus formation that contributes to valvular vegetation in IE. Dabigatran, by inhibiting thrombin, decreases the conversion of fibrinogen to fibrin. Thus, dabigatran might be a potential therapeutic strategy to decrease clotting and fibrin formation, thereby having an effect on decreasing vegetation and embolism. A recent study evaluating the adjunctive role of dabigatran in a murine model of *S. aureus* IE demonstrated dabigatran’s efficacy in reducing vegetation size, inflammation, and bacterial load [23]. Pharmacokinetic details are presented in Table 5 [23,59,60]. Although preliminary studies suggest that dabigatran may reduce vegetation size and the risk of embolism, more research is needed to clarify its clinical utility.

#### 4.1.4. Novel Therapeutic Strategies

Given the challenges posed by biofilm-associated resistance, novel therapeutic strategies are being explored. Various approaches have been proposed for eradicating *S. aureus* biofilms [24,25,26,27,28,29,30,31], which are briefly discussed below.

##### Antimicrobial Peptides (AMPs)

Antimicrobial peptides (AMPs), also known as host defense peptides (HDPs) [27,28], are small molecules with broad-spectrum, rapid antibacterial activity against drug-resistant pathogens and have garnered significant attention as potential adjunctive therapeutics for *S. aureus* infections [61,62,63]. AMPs target biofilms through several mechanisms: destabilizing cell membranes, disrupting bacterial communication, degrading the biofilm matrix, inhibiting bacterial stress response systems, and downregulating genes involved in biofilm formation and protein transport. When combined with antibiotics, AMPs demonstrate even greater efficacy against biofilms. AMPs disrupt the structural integrity of bacterial cell membranes, causing leakage of cellular contents leading to bacterial death [64,65,66,67]. They also inhibit bacterial adhesion by targeting biofilm-associated proteins; for instance, HBD3 and HBD3-derived peptides reduce adhesin gene expression, preventing biofilm formation [68,69,70]. Additionally, AMP LL-37 interferes with biofilm formation by blocking quorum sensing (QS), a bacterial communication system [71]. Notably, SAAP-148 has been shown to rapidly eliminate *S. aureus* biofilms, including antimicrobial-resistant strains, within two hours [72,73].

Although AMPs show great promise due to their in vitro biofilm-inhibiting effects, challenges such as toxicity and poor stability under physiological conditions limit their broader clinical use [74,75].

##### Quorum Sensing Inhibitors (QSIs)

Quorum sensing inhibitors (QSIs) are emerging as potential antibiofilm agents. Studies have shown that QSIs like Hamamelitannin (HAM) can block quorum sensing via the TraP QS system and enhance the susceptibility of *S. aureus* biofilms to antibiotics such as vancomycin [76,77,78]. HAM has been shown to alter cell wall synthesis and extracellular DNA release, increasing biofilm susceptibility. The effectiveness of this QSI has been demonstrated in mouse models in vivo [76]. Additionally, some AMPs also possess QSI properties [71].

##### Bacteriophages

Bacteriophages have the ability to lyse bacterial cells and biofilm extracellular matrix (ECM), even during bacterial dormancy, leading to cell lysis upon reactivation [79]. Phage lysins, such as LysGH15, have shown efficient anti-biofilm activity against *S. aureus* in vitro [27,80]. Bacteriophages are species-specific, with minimal risk of developing resistance, and they selectively target bacterial cells without affecting human cells, resulting in fewer adverse effects [81]. Bacteriophage therapy holds promise as an adjunctive therapeutic option, although further studies are required to determine efficacy, pharmacokinetics, and delivery methods [27].

##### Immunotherapy

The development of vaccines and antibodies against *S. aureus* has been challenging due to the pathogen’s numerous virulence factors. Various vaccine candidates, including capsular polysaccharides (types 5 and 8), fibronectin-binding protein, clumping factors A and B, and others, have been investigated to prevent *S. aureus* infections [82]. It has been proposed that monoclonal antibodies conjugated to antibiotics may concentrate antibiotics at the infection site, thereby enhancing their effectiveness [27,83]. However, these strategies remain largely experimental, and their real-world applicability is yet to be determined.

##### Nanoparticles

Nanoparticles (NPs) have shown antibacterial and antibiofilm properties [26,27,84,85]. Nanomaterials such as gold, silver, lipids (e.g., liposomes), and polymers (e.g., chitosan) have demonstrated antibiofilm activity by disrupting the bacterial membrane, inducing oxidative stress, and interfering with bacterial metabolism [86,87,88,89,90,91,92,93,94,95,96]. For example, titanium dioxide has been shown to hinder EPS formation, and magnesium oxide (MgO) can disrupt bacterial membranes. Rifampicin-conjugated silver nanoparticles (Rif-Ag NPs) have inhibited over 90% of biofilms formed by MRSA at low doses [97]. Under laser irradiation, gold nanoparticles (AuNPs) produce vapor nanobubbles (VNBs) that disrupt biofilm integrity, enhancing antibiotic penetration [88]. Chitosan nanoparticles (CNPs) have also been shown to inhibit biofilm formation and damage mature biofilms [90,98]. While promising, nanotechnology requires clinical validation to verify its real-world applicability [27].

##### Combination of Antibiofilm Agents with Antibiotics

Given that biofilm formation contributes to antibiotic resistance, combination therapy is emerging as a promising therapeutic approach. Some examples include:

Surface Active Agents: Combining antibiotics with agents like N-acetylcysteine and recombinant deoxyribonuclease I (DNase I) has proven effective in inhibiting biofilm formation and improving bacterial eradication [27,99,100]. Other agents, such as catechin and vanillic acids, have shown synergistic effects with antibiotics by reducing bacterial adhesion [101].

Bacteriophage Therapy: Bacteriophages, used in combination with antimicrobials, have shown promise in lysing bacterial cells and biofilm matrices, offering a targeted approach with minimal side effects and may offer a useful option for highly resistant strains [102,103].

Adjunctive Therapies: Hyperbaric oxygen therapy (HBOT) has demonstrated potential as an adjunctive treatment in reducing bacterial load and biofilm size in animal models [24,57]. Hyperbaric oxygen therapy (HBOT) is a well-established but debated treatment involving the administration of 100% oxygen in a chamber pressurized above atmospheric levels (>1 ATA). Although described almost 3 centuries ago, its precise mechanism of action remains unclear. However, it substantially increases dissolved oxygen in plasma, thereby enhancing oxygen delivery to tissues independently of hemoglobin [104]. Biofilms, such as those formed by MRSA, create hypoxic microenvironments that impair host immune responses and diminish antibiotic effectiveness. HBOT has been shown to be a key intervention, as it improves cellular oxygenation within these hypoxic biofilms. In vitro studies demonstrate that HBOT enhances the efficacy of antibiotics by increasing oxygen-dependent antimicrobial activity [105]. The increased oxygen penetration—up to fourfold—into biofilms stimulates bacterial metabolic activity, making the bacteria more vulnerable to antibiotics [57].

Nanoparticle-Based Drug Delivery: Nanoparticle technology is being explored to enhance antibiotic penetration and biofilm disruption, which may improve treatment outcomes for biofilm-associated infections [91].

##### Other Potential Strategies

Other novel strategies are also being explored. For example, cyclic diguanylate (C-di-GMP) exposure has been shown to decrease intercellular adhesion and biofilm formation in *S. aureus* [57,106]. Quaternary ammonium compounds exhibit broad-spectrum activity against biofilms, but their toxicity to human cells is a concern [31,107]. Additionally, ultraviolet light and antibacterial photodynamic therapy (APDT), using photosensitizers like phenothiaziniums, are being investigated, though their current limitations include potential cellular toxicity and limited tissue penetration [31,108,109]. Further studies are needed to assess the clinical relevance of these approaches.

## 5. Conclusions

MRSA-associated IE represents a formidable clinical challenge due to the pathogen’s ability to form biofilms, which significantly complicates its pathogenesis, clinical presentation, and treatment. Biofilm formation allows MRSA to adhere to both native and prosthetic cardiac tissues while shielding itself from host immune defenses and antibiotic treatments. This complex, multifaceted process—driven by microbial surface components, regulatory systems, and interactions with host tissues—underpins the persistent and aggressive nature of MRSA infections, especially in IE.

The clinical manifestations of MRSA-associated IE vary widely, ranging from fever and bacteremia to more severe complications such as embolic events, heart failure, and neurological damage. Early diagnosis remains crucial to improving patient outcomes, and the updated Duke Criteria, which incorporate advanced imaging techniques and consider biofilm-associated infections on prosthetic materials, have enhanced diagnostic accuracy. However, MRSA biofilms on prosthetic devices and heart valves continue to complicate management, often necessitating prolonged antibiotic regimens and frequent surgical interventions. Treatment of biofilm-associated IE is particularly challenging in the context of MRSA, as biofilm formation not only promotes antibiotic resistance but also limits the effectiveness of standard therapeutic approaches. While antibiotics remain the mainstay of treatment, early and aggressive surgical intervention is critical in cases where infection persists or poses an embolic risk. Surgical removal of infected tissues and prosthetic materials remains a cornerstone of effective management in MRSA-associated IE. In addition, novel therapeutic strategies to combat biofilms are emerging. These include antimicrobial peptides, quorum sensing inhibitors, bacteriophage therapy, and nanoparticle-based drug delivery systems, all of which show promise in disrupting biofilm integrity and enhancing antibiotic efficacy. These innovative approaches target biofilm structure and bacterial communication pathways, opening new avenues for treatment. However, further clinical trials are needed to validate the efficacy and safety of these therapies in real-world settings.

## 6. Future Directions

MRSA-associated IE is a complex and evolving clinical problem, with biofilm-mediated resistance posing persistent challenges in both diagnosis and treatment. While current therapeutic regimens are essential, they often require supplementation with surgical intervention, and emerging targeted strategies may offer important additional tools to address the limitations imposed by biofilms. The integration of novel therapies alongside established clinical practices offers a promising future for improving patient outcomes and reducing the high morbidity and mortality associated with MRSA IE. Continued research and innovation in diagnostics and therapeutics are essential for combating MRSA IE, one of the most difficult infections in modern medicine.

## Figures and Tables

**Table 1:** Summary of the pathogenesis of MRSA infective endocarditis.

Phase	Description
Initial Infection and Biofilm Formation	MRSA attaches to host surfaces and begins forming a biofilm.
Attachment Mechanisms	MRSA binds to tissues via damage-induced (using ClfA/B to adhere) or inflammation-induced pathways.
Biofilm Maturation	The biofilm matures, forming a protective matrix that shields MRSA from immune defenses.
Virulence Regulation	Nutrient depletion triggers toxin release, which aids in immune evasion and causes tissue damage.
Immune Response and Cytokine Storm	The immune system escalates, leading to a cytokine storm, inflammation, and tissue damage.
Biofilm Dispersal and Embolization	Fragments of the biofilm break off, spreading infection through emboli, which can block vessels.
Systemic Infection and Immune Evasion	MRSA evades immune defenses, risking septic shock, organ failure, and severe systemic infection.

**Table 2:** Antibiotics for the treatment of Native Valve and Prosthetic Valve Infective Endocarditis in Pediatric and Adult Patients [1,2].

	Adult		Pediatric
	AHA (Updated 2015)	ESC (Updated 2023)	AHA (Updated 2015)
Right-sided and Left-sided Native Valve Endocarditis	First-line *: Vancomycin ^Δ€^	First-line **: Vancomycin ^Δ€^	First-line *: Vancomycin ^Δ€^ ± Gentamicin ^ΔΠ^ 1 mg/kg IV every 8 h × 3–5 days.
	Second-line *: Daptomycin ^Δ^ ≥ 8 mg/kg IV every 24 h	Second-line **: Daptomycin ^Δ^ 10 mg/kg IV every 24 h + Ceftaroline ^Δ^ 600 mg IV every 8 h OR Fosfomycin ^Δ◊^ 2–3 G IV every 6 h OR Cloxacillin 2G IV every 4 h	
Prosthetic Valve Endocarditis	First-line ^#^: Vancomycin ^Δ€^ + Rifampin^Δ∞^ 300 mg IV every 8 h + Gentamicin ^ΔΠ¥^ 1 mg/kg IV every 8 h × 2 weeks	First-line ^#^: Vancomycin ^Δ^ OR Daptomycin ^Δ^ 10 mg/kg IV every 24 h + Rifampin^Δ∞^ 900–1200 mg per 24 h IV divided every 8–12 h. + Gentamicin ^ΔΠ¥^ 1 mg/kg IV every 8 h × 2 weeks	First-line ^#^: Vancomycin ^Δ€^ + Rifampin ^Δ∞^ 5 mg/kg IV every 8 h, max. = 900 mg/day + Gentamicin ^ΔΠ¥^ 1 mg/kg IV every 8 h × 2 weeks

* Continue the regimen for 6 weeks. ** Continue the regimen for 4–6 weeks. ^#^ Continue the regimen for at least 6 weeks. ^Δ^ Requires renal dose adjustment. ^€^ Dosing guidelines updated in 2020 recommend a loading dose of 20–35 mg/kg using actual body weight (ActBW), not to exceed 3000 mg, then 15–20 mg/kg IV every 8–12 h in patients with normal renal function. Goal AUC value = 400–600 mg·h/L is the preferred monitoring method. Alternatively, goal troughs of 15–20 mcg/mL have been utilized. ^Π^ Dose gentamicin based on actual body weight for underweight patients, ideal body weight for non-obese patients, and adjusted body weight for obese patients. ^◊^ Only availabel orally in the United States. ^¥^ Initiate therapy on day 1. Limit therapy to 2 weeks duration to limit toxicities such as ototoxicity and nephrotoxicity. ^∞^ Initiate therapy after 3–5 days of antimicrobial therapy with other agents to reduce bacterial load and stem developing resistance to rifampin.

**Table 3:** Antimicrobials for the treatment of MRSA infective endocarditis [1,2,54].

Medication	Class	Mechanism of Action	Route	Adverse Drug Reactions	Pharmacokinetics
Vancomycin	Glycopeptide	Binds to D-alanyl-D-alanine inhibiting cell wall synthesis and blocking glycopeptide polymerization.	IV, PO, Rectal	Common: Abdominal pain, Diarrhea, Hypokalemia, Nausea/vomiting Serious: Agranulocytosis, Anaphylaxis, *C. difficile* diarrhea, Nephrotoxicity, Neutropenia, Ototoxicity, Thrombocytopenia	Absorption: IV: 100%. Oral/rectal: negligible Distribution: Distributes widely and crosses blood–brain barrier. Vd: 0.2 L/kg–1.25 L/kg Metabolism: None Excretion: IV is renally excreted, PO = fecally excreted unchanged, dialyzable Elimination Half-Life: Adults: 4–6 h, Pediatrics: 5 to 21 h
Rifampin	Rifamycin	Blocks RNA transcription and bacterial RNA synthesis by binding to the beta subunit of DNA-dependent RNA polymerase.	IV, PO	Common: None Serious: Agranulocytosis, Anaphylaxis, Disseminated intravascular coagulation, Hepatotoxicity, Interstitial lung disease, Nephrotoxicity, Thrombotic microangiopathy	Absorption: IV: 100%. Oral: well absorbed, food may delay absorption. Distribution: Crosses blood–brain barrier, lipophilic Vd: 0.66 L/kg Metabolism: Hepatic Excretion: Urine around 30%, feces 60–65%, non-dialyzable. Elimination half-life: Adults: 2–3 h, Pediatrics: 1–4 h
Daptomycin	Cyclic Lipopeptide	Inhibits synthesis of DNA, RNA and protein intracellularly. Causes cell wall depolarization.	IV	Common: Abdominal pain, Diarrhea, Dizziness, Dyspnea, Fever, Headache, Hypertension, Hypotension, Insomnia, Pain in throat, Pruritis, Rash, Vomiting Serious: Increase in creatinine kinase level, Pulmonary eosinophilia, Renal failure, Rhabdomyolysis	Absorption: IV: 100%. Distribution: Distributes widely. Lung surfactants inactivate the drug. Crosses blood–brain barrier. Vd: 0.1 L/kg Metabolism: Insignificant Excretion: 78% renally, 5.7% fecally, dialyzable Elimination half-life: Adults: 8 h, Pediatrics: 4.4–7.5 h
Ceftaroline	Cephalosporin 5th Generation	Inhibits bacterial wall synthesis by binding to penicillin-binding proteins.	IV	Common: Diarrhea, Fever, Nausea/vomiting, Rash Serious: Anaphylaxis, *C. difficile* diarrhea, Elevation in ALT/SGPT level Encephalopathy, Seizure	Absorption: IV: 100% Distribution: Distributes widely Vd: 20.5 L Metabolism: Phosphatase enzyme metabolizes to active drug in plasma Excretion: 88% renally, 6% fecally, dialyzable Elimination half-life: 1–3 h
Linezolid	Oxazolidinone	Inhibits bacterial protein synthesis by binding to the bacterial 23S ribosomal RNA of the 50S subunit.	IV, PO	Common: Diarrhea, Headache, Nausea/vomiting Serious: *C. difficile* infection, Disorder of optic nerve, Hepatic injury, Hyponatremia, Lactic acidosis, Myelosuppression, Peripheral neuropathy, Seizure, Serotonin syndrome, Syndrome of inappropriate antidiuretic hormone secretion	Absorption: IV: 100%. Oral: 100% Distribution: Distributes widely. Crosses blood–brain barrier. Vd: 0.65 L/kg Metabolism: Hepatic Excretion: 80% in urine, 9% fecally as metabolites, dialyzable. Elimination half-life: 5 h
Dalbavancin	Glycopeptide	Binds to D-alanyl-D-alanine inhibiting cell wall synthesis and blocking glycopeptide polymerization.	IV	Common: Diarrhea, Fever, Headache, Nausea Serious: *C. difficile* diarrhea, Elevated ALT/SGPT, GI hemorrhage, Hypersensitivity reaction	Absorption: IV: 100% Distribution: Extensive in skin. Vd: 9 L Metabolism: Negligeable. Excretion: 45% in urine, 20% fecally, nondialyzable. Elimination half-life: 346 h

**Table 4:** Pediatric and adult dosing of anti-MRSA antibiotics for infective endocarditis [1,2,55].

Medication	Adult Dose (MRSA IE Indication Only)	Pediatric Dose (MRSA Indications Only)
Vancomycin	10–20 mg/kg IV every 8 to 48 h in adults. May consider a bolus of 20–35 mg/kg. Requires pharmacokinetic calculator and AUC monitoring to guide the proper dose. Higher AUC (closer to 600) are recommended for patients with endocarditis. Requires renal dose adjustment.	Serious MRSA Infection Treatment: 3 mo–11 yo: 60–80 mg/kg/day IV divided every 6 h (max. dose: 3600 mg/day) 12 yo–18 yo: 60–70 mg/kg/day IV divided every 6–8 h (max. dose: 3600 mg/day) Requires pharmacokinetic calculator and AUC monitoring to guide proper dose. Higher AUC (closer to 600) are recommended for patients with endocarditis. Requires renal dose adjustment.
Rifampin	Endocarditis Synergy (off-label): 300–600 mg IV or PO every 12 h in combination with other antibiotics. May require renal dose adjustment.	Endocarditis Synergy: 1 mo–18 yo: 5 mg/kg IV or PO every 8 h (max. dose: 900 mg/day) May require renal dose adjustment.
Daptomycin	8–12 mg/kg IV every 24 h. Requires renal dose adjustments.	1 mo–5 yo: 10 mg/kg/dose IV every 24 h 6 yo–18 yo: 6 mg/kg/dose IV every 24 h Requires renal dose adjustment.
Ceftaroline	Off-label use: 600 mg IV every 8 h. Requires renal dose adjustment.	Not recommended for endocarditis in pediatrics.
Linezolid	Off-label use: 600 mg IV or orally every 12 h.	Not recommended for endocarditis in pediatrics.
Dalbavancin	Off-label use: 1.5 g IV once, then 500 mg IV once 7 days later. Requires renal dose adjustment.	Not recommended for endocarditis in pediatrics.

**Table 5:** Anticoagulant medications [23,59,60].

Medication	Class	Dosing	Mechanism of Action	Route	Adverse Drug Reactions	Pharmacokinetics
Dabigatran	Direct thrombin inhibitor	No recommended dosing for MRSA IE.	Inhibits thrombin, preventing thrombus development.	Oral	Common: Gastritis, gastrointestinal hemorrhage/ulcer, Hemorrhage Serious: Anaphylaxis, Epidural hematoma, Intracranial hemorrhage, Myocardial infarction	Absorption: Oral: 3–7% capsules, 37% oral pellets Distribution: 50–70 L, 35% protein bound Metabolism: Hydrolyzed to form an active drug Excretion: Primarily renal, dialyzable Elimination Half-Life: 12–17 h
Warfarin	Vitamin K antagonist	0.5–20 mg, variable dose guided by disease-specific INR goals	Reduces synthesis of vitamin K-dependent clotting factors II, VII, IX, and X, as well as proteins C and S.	Oral	Significant: Atheroemboli/cholesterol micro emboli, Calciphylaxis, Reduced bone mineral density, Hemorrhage, Skin necrosis	Absorption: Oral: 100% Distribution: 0.14 L/kg Metabolism: CYP2C9 primarily; CYP2C8, 2C18, 2C19, 1A2, and 3A4 to a lesser extent Excretion: Urine: 92% Elimination Half-Life: 20–60 h

## Data Availability

No new data were created or analyzed in this study.

## Appendix A

Box A1 Definition of infective endocarditis (IE) according to the Modified Duke Criteria per AHA/IDSA guidelines [1].

Definite IE:

- Pathological Criteria:
  ○ Microorganisms confirmed by culture or histological examination of a vegetation, embolized vegetation, or intracardiac abscess specimen.
  ○ Pathological lesions, such as vegetation or intracardiac abscess, confirmed by histological examination showing active endocarditis.

- Clinical Criteria:
  ○ Two major criteria,
  ○ One major criterion and three minor criteria, or
  ○ Five minor criteria.

Possible IE:

- One major criterion and one minor criterion, or
- Three minor criteria.

Rejected IE:

- An alternative diagnosis explaining the symptoms of IE,
- Resolution of IE symptoms with antibiotic therapy for ≤4 days,
- No pathological evidence of IE during surgery or autopsy following ≤4 days of antibiotic therapy, or
- Does not meet the criteria for possible IE as outlined above.

Box A2 Definition of terms used in the Modified Duke Criteria for diagnosing IE per AHA/IDSA guidelines [1].

Major Criteria:

- Blood Culture Positive for IE:
  ○ Typical microorganisms consistent with IE from two separate blood cultures, such as *Viridans streptococci*, *Streptococcus bovis*, HACEK group, *Staphylococcus aureus*, or community-acquired enterococci in the absence of a primary focus.
  ○ Microorganisms consistent with IE from persistently positive blood cultures, defined as at least two positive cultures drawn > 12 h apart, or three of four separate cultures with the first and last samples drawn at least 1 h apart.
  ○ Single positive blood culture for *Coxiella burnetii* or anti-phase 1 IgG antibody titer ≥ 1:800.

- Evidence of Endocardial Involvement:
  ○ Echocardiogram positive for IE (TEE recommended for patients with prosthetic valves, rated at least possible IE by clinical criteria, or complicated IE with paravalvular abscess).
    ■ Findings include an oscillating intracardiac mass on the valve or supporting structures, in the path of regurgitant jets, or on implanted material without an alternative anatomic explanation.
    ■ Presence of an abscess or new partial dehiscence of a prosthetic valve.
  ○ New valvular regurgitation (worsening or changing pre-existing murmur).

Minor Criteria:

- Predisposition: Presence of a predisposing heart condition or intravenous drug use (IDU).

- Fever: Temperature > 38 °C (100.4 °F).

- Vascular Phenomena: Major arterial emboli, septic pulmonary infarcts, mycotic aneurysm, intracranial hemorrhage, conjunctival hemorrhages, and Janeway lesions.

- Immunological Phenomena: Glomerulonephritis, Osler nodes, Roth spots, and positive rheumatoid factor.

- Microbiological Evidence: Positive blood culture that does not meet major criteria (excluding single positive cultures for coagulase-negative staphylococci and other organisms not typically causing endocarditis) or serological evidence of active infection with an organism consistent with IE.

  Note: HACEK refers to a group of fastidious Gram-negative bacteria that are part of the normal flora of the oral-pharyngeal region. The group includes *Haemophilus* species, *Aggregatibacter* species, *Cardiobacterium hominis*, *Eikenella corrodens*, and *Kingella* species. These organisms are less commonly associated with infective endocarditis but can be significant pathogens, particularly in cases where blood cultures are persistently negative. Due to their slow-growing nature, infections caused by HACEK organisms can present diagnostic challenges, often requiring extended incubation periods for cultures. When identified, HACEK organisms typically respond well to beta-lactam antibiotics, including ceftriaxone, which is the recommended first-line treatment. Early identification and appropriate antibiotic therapy are crucial for managing endocarditis caused by these organisms.  IDU, injection drug use; IE, infective endocarditis; IgG, immunoglobulin G; TEE, transesophageal echocardiography; and TTE, transthoracic echocardiography.
